# A roadmap for sex- and gender-disaggregated health research

**DOI:** 10.1186/s12916-023-03060-w

**Published:** 2023-09-13

**Authors:** Sanne A. E. Peters, Mark Woodward

**Affiliations:** 1 Julius Center for Health Sciences and Primary Care, University Medical Centre Utrecht, Utrecht University, Utrecht, The Netherlands; 2 School of Public Health, The George Institute for Global Health, Imperial College London, London, UK; 3 The George Institute for Global Health, University of New South Wales, Sydney, Australia

**Keywords:** Sex, Gender, Research methods, Epidemiology, Health research

## Abstract

Sex and gender are fundamental aspects of health and wellbeing. Yet many research studies fail to consider sex or gender differences, and even when they do this is often limited to merely cataloguing such differences in the makeup of study populations. The evidence on sex and gender differences is thus incomplete in most areas of medicine. This article presents a roadmap for the systematic conduct of sex- and gender-disaggregated health research. We distinguish three phases: the exploration of sex and gender differences in disease risk, presentation, diagnosis, treatment, and outcomes; explaining any found differences by revealing the underlying mechanisms; and translation of the implications of such differences to policy and practice. For each phase, we provide critical methodological considerations and practical examples are provided, taken primarily from the field of cardiovascular disease. We also discuss key overarching themes and terminology that are at the essence of any study evaluating the relevance of sex and gender in health. Here, we limit ourselves to binary sex and gender in order to produce a coherent, succinct narrative. Further disaggregation by sex and gender separately and which recognises intersex, non-binary, and gender-diverse identities, as well as other aspects of intersectionality, can build on this basic minimum level of disaggregation. We envision that uptake of this roadmap, together with wider policy and educational activities, will aid researchers to systematically explore and explain relevant sex and gender differences in health and will aid educators, clinicians, and policymakers to translate the outcomes of research in the most effective and meaningful way, for the benefit of all.

## Background

Sex and gender are fundamental drivers of virtually all major causes of death and disease [1]. Despite this, evidence informing today’s health care policies and practices is still largely obtained from predominantly male study populations. It is assumed that the evidence from these ‘male studies’ is equally applicable to women. However, this is not necessarily true, and sex- and gender-inclusive health research is vital to improve health outcomes for both women and men.

The importance of sex and gender as a routine part of research has led to policy changes at major funding agencies, worldwide [2, 3]. Despite significant uptake and growing awareness for sex- and gender-inclusive research and reporting, critical barriers remain. Indeed, women continue to be underrepresented in clinical trials in various domains and sex- and gender-disaggregated analyses and reporting, including on gender-diverse participants, are still frequently omitted, often without justification [4–10].

 While guidelines and policies are one way to change research practices, it should not end up being a checkbox exercise. Where sex- and gender-disaggregated analyses are conducted, they are frequently mainly descriptive in nature. Whilst knowing where sex and gender differences exist (and where not) is important, it is only a necessary first step in the research and translation cycle. Equipping the research community with the necessary skills and knowledge to embed sex and gender considerations as a routine part of their work should lead to a more systemic change at the grassroot level.

In this article, we present a roadmap for the conduct of sex- and gender-disaggregated research with the aim to further increase their uptake, scope, and quality. We explain the roadmap with examples, mainly drawn from the field of cardiovascular disease (CVD), and provide practical recommendations on how to improve sex- and gender-disaggregated health research.

## Main text

### Defining sex and gender

Sex and gender are integrally related and influence health in different ways [11]. According to the World Health Organization, sex refers to ‘the different biological and physiological characteristics of females, males and intersex persons, such as chromosomes, hormones and reproductive organs’, whilst gender refers to ‘the socially constructed characteristics of women, men, girls and boys’. This includes norms, behaviours and roles associated with being a woman, man, girl or boy, as well as relationships with each other. These can vary from society to society and can change over time. The gender construct can be described in three related dimensions; gender norms, gender identity, and gender relations that together encompass the socially constructed roles, relationships, behaviours, relative power, and other traits that societies routinely ascribe to women and men [12]. A more comprehensive definition, also inclusive of diverse genders, refers to gender as follows: ‘depending on the context, gender may reference gender identity, gender expression, and/or social gender role, including understandings and expectations culturally tied to people who were assigned male or female at birth’. Gender identities other than those of men and women (who can be either cisgender or transgender) include transgender, nonbinary, genderqueer, gender neutral, agender, gender fluid, and ‘third’ gender, among others; many other genders are recognized around the world’ [13].

Decision trees for the steps involved in analysing and reporting sex versus gender have been described before [14]. In this article, we will speak of women and men throughout, instead of females and males, as we feel this is more holistic and reflective of the complex interplay of sex and gender factors on human beings. We acknowledge that this is a simplification of the reality as sex and gender are often intertwined, especially when studying behavioural or societal factors. We also acknowledge that the dichotomy of women and men does not cover the true non-binary nature of both sex and gender and that this approach might not be sufficient for research across different gender identities [12, 15, 16]. However, this choice does not affect the roadmap presented here, as the principles remain the same. Since studies typically do not separate between sex and gender, and few studies have considered sex (or gender) beyond binary variables (although numbers are rightly increasing) [17], our approach is consistent with the majority of medical and health research. Taking such an approach does not affect the essence of our roadmap, as the phases that we present later remain the same. However, the methodologies within each phase need minor adaptations when a holistic approach is taken to account for sex and gender diversity.

### Intersectionality

We limit this article to sex and gender differences, acknowledging that not all women are the same, and neither are all men. Ideally, research should explore differences in an intersectional perspective, including combinations of socio-demographic features [18]. For instance, women’s risk for coronary heart disease (CHD) is one third of that of men overall, but this statement hides the fact that the sex ratio decreases with increasing age and differs across regions [19]. However, in human populations, sex or gender is generally split in roughly equal numbers, or at least data are more equally distributed by sex and gender than by other socio-demographic factors. So, we argue that disaggregation by sex or gender, or both, is a minimum requirement for health research. Researchers should determine themselves whether sex, gender, or both, are most relevant to their research and disaggregate the research accordingly. We leave it to others to make similar arguments to those expressed here for other important aspects of intersectionality.

### What is meant by sex- and gender-disaggregated research

Health and medical studies often include a diverse group of people, including people that differ by sex and gender. The published article may well report differences in the study population by sex or gender, typically in a table with baseline characteristics (i.e. what is often called ‘Table 1’). They may also go on to adjust, or ‘control’, their study outcome results for sex or gender, either by fitting multivariable regression models or weighting sex/gender results equally or according to their distribution in the parent population. Neither analysis can be claimed as ‘taking account of sex and gender’. Sex- and gender-disaggregated research requires the outcomes, not the inputs, to be reported and interpreted by sex and/or gender and thus, rather than remove the effects of sex and gender on outcomes, show what differences there are in such effects. Only in this way can important questions, such as whether a new drug is equally effective in women and men, be resolved.

### Sex- and gender-disaggregated research is not only about women

Another common misperception is that sex- and gender-disaggregated research only benefits women. Sex- and gender-disaggregated research initially aimed to address the lack of research on women in many disease areas and the assumption that men’s patterns of disease apply to women. The primary group expected to benefit are indeed women. However, sex- and gender-disaggregated research also benefits men, boys, and girls. Men, for example, have been largely neglected in osteoporosis or rheumatoid arthritis, as it is considered a disease of older women. Changing the name of organisations and initiatives for sex- and gender-specific research and medicine to include ‘women and men’ rather than just ‘women’ in their name would help dispel this notion. Also, a greater representation of male researchers in this research area would help to redress this misperception. Ultimately, this kind of research is not only about women—it is about getting the science right for the benefit of all.

### Using the other sex as a comparator group

Studies that only involve one sex are clearly appropriate when the exposure (i.e. risk factor) of interest can only apply to that single sex. However, many studies are carried out only in women, or only in men. Whilst these certainly can provide useful evidence of effects for that sex, their interpretation is inevitably limited, whilst reports of findings may be misleading (Table 1). Using the other sex as a comparator group can also help to disentangle mechanisms, for example involving reproductive processes that only affect one sex.

**Table 1:** Using the other sex as the comparator to put finding into a perspective and disentangle mechanisms

Example 1: Finding that a large percentage of women do not receive guideline-based care may be headline grabbing, but if men have a similarly low prevalence, the most crucial finding is that better care is required per se. This was the case in a survey of care given to people living with CHD that found only 6% of women were treated to target, for a cluster of risk factors [20]. This is an extremely poor result, which is worthy of attention, but cannot be used to show that women are disadvantaged since the equivalent result for men was 8%. The message here is to, whenever possible, include the other sex, perhaps only to serve as a comparator group, to produce meaningful findings even if the interest of the research is on a single sex
Example 2: As an example of where including men as comparator group led to a different interpretation, consider the effect of increasing family size on cardiometabolic risk. Several studies showed that women with a higher number of pregnancies were at a higher risk of cardiometabolic diseases [21–23]. While there are biological reasons to support this, even when ruling out the role of adverse pregnancy outcomes, having large families might also impose a burden on the cardiovascular system. Men cannot get pregnant, but they do get children. Men can therefore be used as a control group in determining whether it is childbearing or childrearing that explains the associations between the number of pregnancies and cardiovascular risk seen in women. In analyses in the UK Biobank and China Kadoorie Biobank, we demonstrated that the association between number of children and the risk of cardiometabolic diseases was similar in women and men [23–25]. Hence, it may be mainly childrearing, and not childbearing, that underpins the association between the number of pregnancies and cardiovascular risk in women. Interestingly, in the UK Biobank, those with the lowest risk of CVD, had two children whereas having one child was associated with the lowest risk in the China Kadoorie Biobank. This might suggest that societal norms, structures, and policies on preferred family size might explain why those deviating from that preferred standard are at a higher risk of CVD

## Elements of sex and gender-disaggregated research methods

**Fig. 1:**
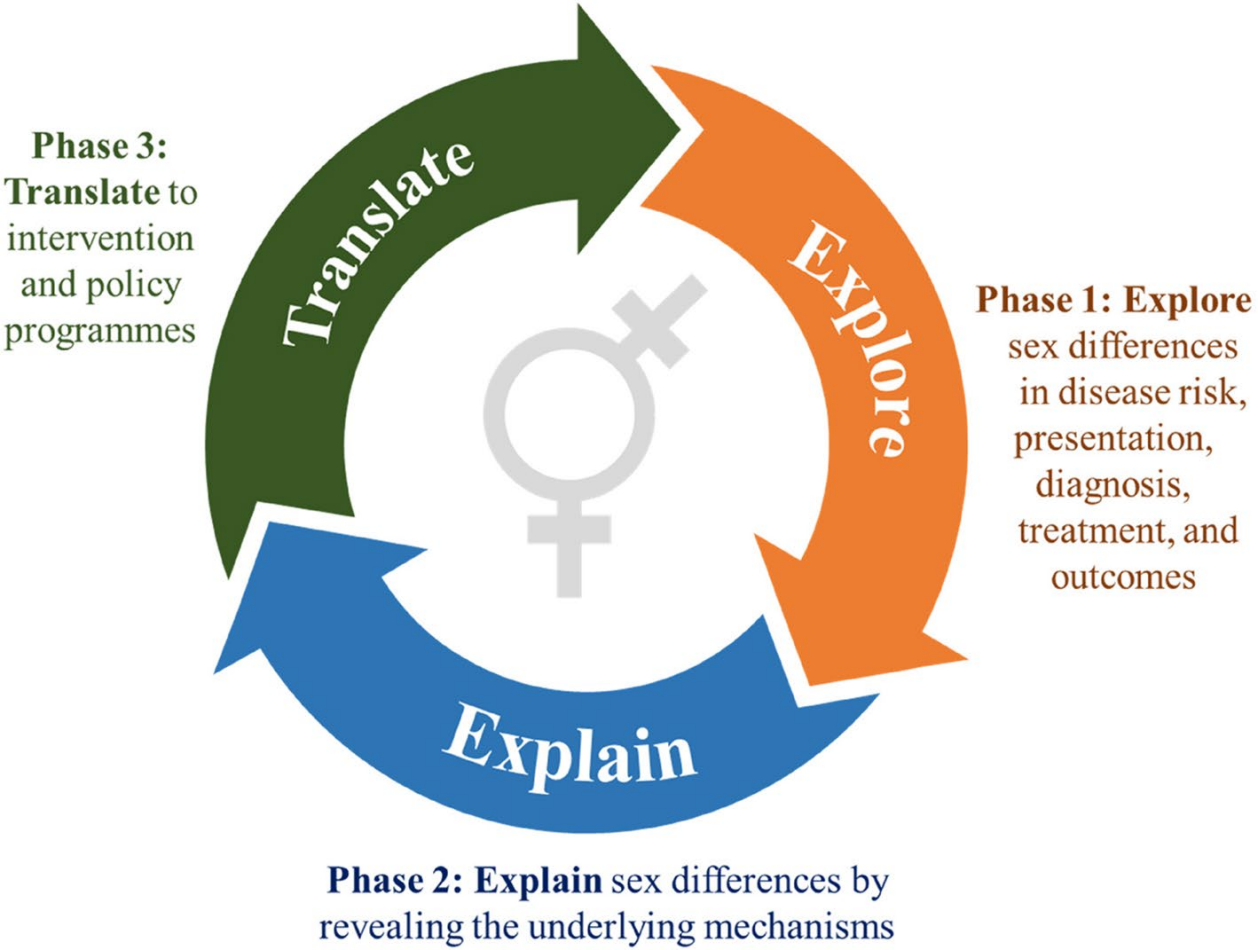
Roadmap for sex and gender-disaggregated research. The three phases in the roadmap for sex- and gender-disaggregated research. The design, analysis, and reporting aspects from Fig. 1 are an integral part of phases 1 and 2, and, in some instances, also of phase 3

**Fig. 2:**
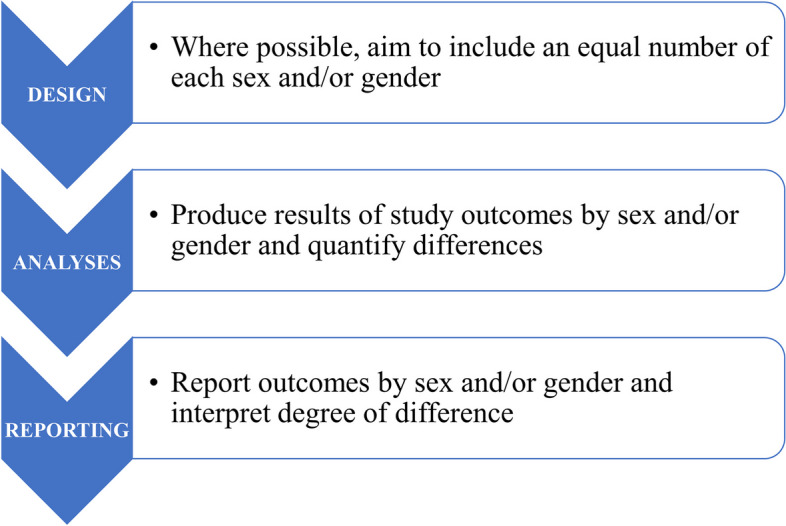
Sex and gender considerations in the design, analysis, and reporting of research. Where the nature of the target population, or the funding mechanism, does not allow for equal numbers some attempt should still be made to recruit a substantial number in each sex and gender group. Where differences in outcomes are unimportant, or space limitations preclude detail when publishing, this should be commented upon, and stratified results included in a web supplement to inform potential future meta-analyses

Sex and gender differences in health arise at many points in the lifespan, often identifiable at episodes of engagement with health systems. Figure 1 illustrates our proposed roadmap for sex-and gender-disaggregated research. Table 2 summarises the key recommendations and Table 3 highlights the strengths and limitations. The roadmap consists of three distinct phases: exploration of sex and gender differences; explanation of sex and gender differences; and translation of sex and gender differences to policy and practice. Adhering to the steps for integrating sex and gender in the design, analysis, and reporting of research as described in Fig. 2 is essential in using the roadmap.

**Table 2:** Key recommendations of the roadmap for sex- and gender-disaggregated health research

Phase 1: Exploration of sex and gender differences - Identify where sex and gender differences do (and do not) exist; - Always report sex-specific findings (with measure of variability); - Do not make conclusions on the presence (or absence) of sex differences based only on the sex-specific findings; - Quantify sex differences using a full interaction model that accounts for the possibility of sex-specific confounding
Phase 2: Explanation of sex and gender differences - Exclude the artefactual explanation; - When evaluating sex differences in the associations of risk factors, consider both the absolute (risk difference) and relative (risk ratio) scales - Assess to what extent any sex or gender differences are due to differences in biology or due to different interactions with the healthcare system; - Use sex-specific Mendelian randomisation to strengthen sex-differentiated causal inferences; - Broaden the scope of research on the role of sex hormones
Phase 3: Translation to policy and practice - Embed sex- and gender-inclusive medicine in the curriculum of health professionals; - Consider including sex-specific recommendations in guidelines;
Systemic factors - Ensure that the participation of women and men in clinical trials, and medical research more broadly, is commensurate with the prevalence of the disease of interest in the population; - Funders and publishers of medical research should make the integration of sex and gender a requirement for funding or publishing; - Enhance the diversity in teams in research, policy, and practice, and address implicit biases against women

**Table 3:** Strengths and limitations of the roadmap

*Strengths*	*Limitations*
The roadmap: - In three distinct phases, allows for a systematic evaluation of sex and gender differences in health and disease; - Provides practical guidance for researchers, policy makers, clinicians, and educators on how to explore and explain sex and gender differences in health and how to translate such findings to policy and practice; - Is generic and can be applied to a broad range health research areas; - Can be adopted to assess other aspects of intersectionality and gender identities	The roadmap: - Underscores that sex and gender exist along a continuum and are often intertwined, yet presents sex and gender as binary variables, to enhance coherence and accessibility; - Does not address the issue of how research into sex might differ from research into gender, or how the two might be researched together; - Has a quantitative focus without discussing the complex cultural and psychosocial concepts underpinning sex and gender; - Is a guiding document, which needs to be adapted to the research question and setting, or translational aim, at hand

### Phase 1: Exploration of sex and gender differences

A critical first step in sex- and gender-disaggregated research is to explore where sex differences occur – as an agent for change towards improved health outcomes. Such exploration leads to the identification of areas where sex and gender differences do, or do not, exist. While there is often a tendency to mainly describe areas where differences are found, identifying and reporting where there are no differences is just as important. Routine conduct and reporting of sex-, and where possible, gender-stratified analyses, even when there is no specific hypothesis, allows researchers and users of research to interpret sex and gender differences in the context of the similarities. Areas in which sex and gender differences are commonly explored are plentiful, and include, but are not limited to, the identification of differences (and similarities) in the following categories.

#### Disease risk and prognosis

The leading causes of death are similar in women and men, which include cardiovascular disease, cancers, and lung diseases [26]. These figures, however, mask sex differences in disease risk across the life span. For example, in adolescence and young to middle-aged adulthood, self-harm and violence and road injuries, respectively, are the number two and three leading causes of death in men whereas HIV/AIDS and sexually transmitted infections are the number three leading cause of death in women. Cardiovascular diseases (number one in men and number two in women) and neoplasms (number one in women) complete these figures for people aged 15–49 years. Women have a longer life expectancy than men, but spend more time in ill-health. For example, women constitute the majority of the residents of nursing homes and two-thirds of individuals living with dementia are women.

#### Disease presentation and diagnosis

Timely and adequate diagnosis is the cornerstone for optimal treatment and management. A study in patients with an acute myocardial infarction showed that women were considerably more likely than men to receive another initial diagnosis, which was associated with lower use of guideline-recommended care and worse outcomes [27]. Such diagnostic delay might be explained by sex differences in symptom presentation. Recent meta-analyses have shown that symptoms at presentation of CHD and stroke can be different between women and men [28, 29]. However, campaigns to raise awareness of heart disease and stroke symptoms are typically based on the male pattern of disease. Lack of awareness of the risk of cardiovascular disease in women [30], including related warning signs, in both patients and care givers, as well as differences in disease aetiology, might also explain the diagnostic delay and misdiagnosis of cardiovascular disease in women.

#### Risk factor prevalence and associations

Sex differences in risk factors essentially manifest at two levels; differences in prevalence and differences in magnitude of the risk factor association. Sex differences in risk factor prevalence relate to the portion of women or men exposed to a risk factor, which can differ over time, between settings, and by age. Female-specific risk factors, such as pregnancy-related factors and factors related to the reproductive lifespan, affect virtually all women. On the other hand, prostate cancer affects only a proportion of all men, and even then, typically in older age groups. Other risk factors are male- or female-dominated. For example, in some parts of the world, large portions of men are smokers or consume alcohol whereas women largely abstain from these unhealthy habits [31]. Yet, women are more often exposed than men to abuse and intimate partner violence and have lower levels of health literacy. There is also a range of shared risk factors, such as hypertension, dyslipidaemia, diabetes, obesity, unhealthy diet, or a sedentary lifestyle, that are common in both women and men.

Sex differences in risk factor associations relate to differences in the strength of the association between the risk (or protective) factor and a disease outcome (Table 4). A detailed tutorial on how to assess sex differences in risk factor associations is provided elsewhere [32].

**Table 4:** Sex differences in the association between diabetes and myocardial infarction

Diabetes is an important risk factor for a range of CVDs, regardless of sex. However, studies have consistently shown that the magnitude of that association in stronger in women [33]. Specifically, analyses in the UK Biobank showed that the adjusted hazard ratio for myocardial infarction associated with type 2 diabetes was 1.96 (1.60; 1.83) in women and 1.33 (1.18; 1.51) in men [34]. The corresponding women-to-men ratio of hazard ratios, as a measure of sex differences, was 1.47 (1.16; 1.87). In other words, the myocardial infarction conferred by diabetes is 47% greater in women than men. However, in absolute terms, the rates of myocardial infarction at a given age are lower in women than men, also in the presence of diabetes. Women lose some of their advantage, in terms of the risk of myocardial infarction, but do not surpass men	

#### Safety and efficacy of interventions

Between 1997 and 2000, ten drugs were withdrawn from the US market because of serious side effects; eight posed greater risks for women than for men [35]. The fact that these data date back to over 25 years ago, and we are unaware of updates, typifies the dearth of evidence regarding sex differences in the safety and efficacy of interventions. Randomised controlled trials are typically not designed to assess sex-specific drug effects, neither in terms of efficacy nor safety. Meta-analyses of previous trials have addressed this issue of power to some extent [36, 37], but issues of limited representativeness and the evaluation of only a selection of outcomes remain. Nevertheless, recent studies using observational data suggest that women might achieve their maximum treatment benefit at a lower drug dosage than men [38, 39]. These sex differences in optimal treatment or treatment intensity could be explained by sex differences in pharmacokinetics or pharmacodynamics, amongst others [40]. For example, given that women generally have a lower bodyweight, higher proportion of body fat, and lower plasma volume, the duration of action of lipophilic (i.e. fat-soluble) drugs may be longer and the peak plasma concentrations of hydrophilic (i.e. water-soluble) drugs may be higher in women. The sedative Ambien is the only drug on the market for which the FDA has different suggested doses based on sex, even though many other drugs are also metabolised differently by men and women.

#### Provision and utilisation of healthcare services

In general, individuals at high risk of a disease and those with established disease require intensive risk factor control. For CHD, there is overwhelming evidence for the effectiveness of drug therapy and lifestyle modification, and hence such measures are universally recommended in clinical guidelines [41]. However, such evidence is often not sex-specific, which can lead to decisions based on personal beliefs or preferences and variation in treatment between the sexes that is not underpinned by guideline recommendations.

#### Quantification of sex differences

The phase of exploring sex and gender differences leads to the identification of areas where sex differences do or do not exist. This phase should also include a formal quantification of the sex-specific results, as well as their corresponding differences. Sex-specific subgroup analyses should be pre-defined and performed, whenever possible and appropriate. The methods for quantifying sex differences, as with any study, depend on the research question at hand. However, some general principles apply, as listed below, and discussed elsewhere [32].

1. Sex-specific results should always be reported. Studies may find no important sex differences or may be powered insufficiently to reliably quantify the presence, or absence, of sex differences. Null results are equally informative and should be reported to avoid publication bias and to be available for inclusion in future meta-analyses.
2. A statistically significant result in one sex but not the other is no evidence for a sex difference. Such a scenario can occur even when the effect estimates are identical between the sexes, but the level of precision of the estimate in one sex is much greater than that in the other (i.e. a wider vs. narrower confidence interval). Such a scenario is likely in several medical disciplines, because women tend to be underrepresented in clinical trials, the gold standard for establishing causality.
3. Always avoid sex-specific conclusions without statistical evidence of an interaction. Event rates can be different between women and men. As statistical power to find an effect, and the corresponding width of the confidence interval for the effect size, increases with an increasing number of events, there is a greater chance of finding an effect in the group with the higher event rate [42]. Many studies have tended to exaggerate the evidence for sex differences by ignoring this fundamental principle.
4. Assessing the sex interaction should not only be based on a *p*-value. It is more meaningful to estimate the sex interaction, together with an accompanying measure of uncertainty, such as a 95% confidence interval. Interaction terms between sex and any potential confounders should also be added to the model for sex differences in the impact of potential confounders on the association under study (i.e. sex-specific confounding).

### Phase 2: Explanation of sex and gender differences

To date, most studies on sex and gender differences have focussed on phase 1; the exploration of the presence or absence of such differences. While critical in identifying and quantifying differences and similarities, studies in phase 1 do not provide explanations for such differences. As such, it often remains unclear what mechanisms, biological or otherwise, underpin the sex differences. Such knowledge is critical to know what could be done about them. Some differences might be an inherent consequence of nature, whereas others represent a sex bias that can, and should, be avoided. Categories that should be considered in explaining any identified sex differences are the artefactual explanation, the accessibility explanation, the biological explanation, and their combination.

#### The artefactual explanation

By artefactual explanations, we mean results that are merely a result of the way studies were designed or analyses have conducted. For example, interview questions might be routinely interpreted in a different way by women and men or a study in which questions are designed by men might be answered less accurately by women.

One might also think that sex differences in the association between some risk factors and disease outcomes, to women’s disadvantage, are a mathematical artefact, explained by the lower ‘background’ risk in women for many diseases (Table 5). But such discordance is not inevitable. For example, recent analyses in the UK Biobank showed that diabetes, smoking, and high blood pressure, but not BMI and blood lipids, were associated with a greater relative risk of CHD in women than men [34, 43]. Hence, sex differences in relative risks are not a mathematical artefact inevitably caused by the lower baseline risk in women. This illustrates that, when evaluating sex differences in the associations of risk factors, it is important to consider both the absolute (risk difference) and relative (risk ratio) scales [32, 44].

**Table 5:** The artefactual explanation

Suppose that the 10-year disease risk in the absence of a risk factor (i.e. the reference group) is 1% in women and 3% in men. In other words, women have a third the risk of men, which — as mentioned already — broadly is the case for CVD (although attenuating with age). When the risk in those with the risk factor is 1% higher in both sexes, this results in a relative risk of 2/1 = 2 in women and of 4/3 = 1.33 in men. That is, women have a 2/1.33 = 1.5 times higher excess risk compared to men when they have the risk factor, even though the risk factor increases the risk by the same amount in both sexes. Thus, some would conclude that this implies that a finding of a higher relative risk in women is purely an artificial finding due to the lower background risk in women and the mathematical (statistical) metric used to compare the sexes	

#### The accessibility explanation

By the accessibility explanation, we mean that women and men may experience diseases differently because their interaction and experience with the health care system are different. Sex differences in disease prevention, treatment, and diagnosis might therefore explain the sex differences in disease risk and outcomes. The sex and gender of the health care provider have also been shown to influence processes and outcomes of care [45–47].

Before we describe some areas where differences exist, it is important to note that, for both women and men, substantial gaps exist between guideline-recommended care and care delivered. In CVD, for example, a large proportion of individuals do not receive the guideline-recommended treatments and do not meet the treatment targets, both in the primary and secondary prevention [20, 48]. This leads to a substantial disease burden, in both women and men, potentially avoidable through more timely diagnosis and better treatment. Several studies have found that women are even less likely than men to be screened regularly, to receive an adequate diagnosis, to be treated according to the clinical guidelines, and to achieve risk factor control [48–53], leading to worse outcomes [54, 55].

##### Sex differences in treatment: appropriate or inappropriate?

Clinical guidelines rarely provide sex-specific treatment recommendations. Differences in treatment in clinical practice are therefore often seen as suboptimal treatment, However, inherent sex differences in the safety and efficacy of medications, or differences in comorbidities and polypharmacy, may be other (appropriate) reasons to treat women and men differently. The question on as to whether women and men might benefit from different treatments has yet to be answered.

Randomised controlled trials (RCTs) are the gold-standard design to study treatment effects. However, they are also conducted in highly selected populations, often with great underrepresentation of women and gender-diverse groups, and are not powered to uncover sex or gender differences [10]. As such, it remains uncertain whether some of the sex differences in treatment, as seen in clinical practice, are explained by inherent differences in drug safety and efficacy. Research in heart failure patients, for example, showed that women reach their maximum treatment effect at a lower dose than men [38, 39]. This sex difference in optimal treatment dosage may be attributable to sex differences in pharmacokinetics, for example, driven by the notable sex differences in body size and composition [40]. Sex differences in treatment may also be justified if the effects of risk factors, as described above [34], are causally different between the sexes. Hence, although there may be avoidable excess treatment gaps in women, some sex differences in treatment may be medically justifiable, yet, not reflected in clinical guidelines. Further research using different study designs with different strengths and limitations is needed to investigate whether women and men achieve better health outcomes if they receive different treatments. Where possible, this should also include investigation of drug effects within subgroups of women and men with, for example, different body sizes.

This issue is not only relevant for drug treatments. For instance, a recent study showed that the accuracy of non-invasive blood pressure measurements, which were lower than invasive measurements, was considerably lower in women than men [56], which might lead to underdiagnosis of hypertension and unrecognised undertreatment. Unless an appropriately large number of both women and men are included in studies, compelling evidence of a sex difference will never be available. On the reverse side, it is equally true that lack of appropriate sex-stratified data, in the cases where a drug has both a similar efficacy and risk in both sexes, can lead to loss of healthy life or death when cautious physicians, with good intentions, deny guideline-based care to those they perceive as more vulnerable. This may explain the lower uptake of guideline-based high-intensity statins after a myocardial infarction in women, compared to men, in the USA [51].

#### The biological explanation

By the biological explanation, we mean that sex differences in health may be explained by inherent biological differences. Women and men are biologically different in terms of genetics, body features, genitalia, and hormones. In addition to differences on the sex chromosome (XX in women and XY in men), women and men also differ considerably on the twenty-two autosomal chromosomes. Indeed, a study in 450,000 individuals of European ancestry in the UK Biobank showed that whilst widespread sex differences exist in genetic architecture for health-related traits, most were modest in magnitude [57]. Other studies found that gene expression and genetic co-expression are influenced by sex in about 30% of tissues [58, 59], thereby providing a biological basis for explaining any sex differences when found.

Most notable are the sex differences in the effects of genetic variants related to body anthropometry. Women and men, on average, have a different body composition and body fat distribution, with women having a higher fat mass and more subcutaneous fat, which results in the characteristic pear-like body shape. Several genome-wide association studies (GWAS) have shown that genetic associations of measures of adiposity strongly differ between the sexes [60–62]; including waist-to-hip circumference, where genetic variants are primarily identified in females.

While the number of sex-stratified GWAS is rising, many still use sex-combined models. This approach could mask potentially relevant genetic variants when these have a differentially signed genetic effect in each sex. That is, a genetic variant could have a positive effect in one sex and a negative effect in the other sex. Combined GWAS analyses could result in a weighted average genetic of near zero, leading to the conclusion of no effect. Masking could also happen when a genetic variant has a large effect in one of the sexes and a small or no effect in the other. In both cases, the weighted average is clearly misleading for both women and men.

##### Sex-specific Mendelian randomisation to strengthen sex-differentiated causal inferences

Mendelian randomisation (MR) is a powerful method to strengthen causal inferences on sex differences in risk factor associations [63]. MR studies exploit the random assortment and independent inheritance of genetic variants in the population, which removes bias due to reverse causation and greatly reduces bias from residual or unmeasured confounding. In MR, single-nucleotide polymorphisms (SNPs) are used as proxies, i.e. instruments, for the exposure of interest. The SNPs that influence the exposure are randomly allocated at meiosis, thus producing a population genotype distribution which is unrelated to the potential confounders an individual is exposed to throughout life. In this regard, MR is comparable to a RCT, where instead of random assortment of genetic variants, individuals are randomly assigned to different therapeutic arms.

By far most MR studies conduct sex-combined analyses, thereby ignoring reported sex differences in the effects of genetic variants on disease phenotypes. A main barrier for sex-specific MR is the limited public availability of sex-specific GWAS results. However, sex-specific MR studies have provided novel insights in the sex-specific effects of certain risk factors on disease outcomes (Table 6) [64–66]. MR can also be used to assess sex differences in the efficacy and safety of drug treatments [67]. Virtually all drug targets are proteins. GWAS have corroborated known effects of licensed drugs through associations at the loci of the genes coding for their corresponding target proteins [68]. By using the genes encoding drug target proteins as instrumental variable for the drug of interest, sex-specific drug-target MR can investigate the sex-specific efficacy and safety of existing drugs, as well as for the identification of new drug targets.

**Table 6:** Sex-specific Mendelian randomisation to strengthen causal inferences

A sex-specific Mendelian randomisation study based on data from the UK Biobank found no sex difference for the strength of the causal effect of genetic liability to type 2 diabetes on the risk of CHD [66]. This was in contrast with strong evidence from observational studies that consistently found evidence for a stronger association in women than men [34]. Another sex-specific Mendelian randomisation study showed that the genetically determined effect of BMI on the risk of type 2 diabetes was stronger in women than men [64]. It may therefore be that the sex differences in the association between diabetes and cardiovascular disease risk seen in observational studies actually occur before the actual diagnosis of diabetes. However, whether causal or otherwise, the higher excess risk seen in women with diabetes suggests a closer eye needs to be kept on them, and shows the importance of sex-specific risk scores	

##### Broaden the scope of research on the role of sex hormones

The sex hormones, oestrogen and testosterone, play an important role in both reproductive and non-reproductive systems. The contribution of hormones to understanding sex differences in health and disease, however, remains debated. To date, most research has focused on the role of oestrogen, which is thought to have an important role in the cardiovascular system, as it has vasodilator effects and reduces or prevents platelet activation [69]. In addition, it improves the profile of circulating lipoproteins, modulates blood pressure, and may underpin the observed sex differences in arterial blood pressure and differences in blood pressure between premenopausal versus postmenopausal women.

Studying the role of sex hormones in women is challenging, given the complexity of accurately measuring natural levels during women’s monthly cycle. A recent study in the UK Biobank showed that the presumed cardioprotective effects of oestradiol seem to be largely confounded by age [70]. Early menopause in women, as a marker of accelerated reproductive ageing, has been associated with a higher risk of CHD and stroke in observational studies. However, the presumed adverse effects of an early menopause on cardiovascular risk have also been brought into question by new evidence from a MR study, which showed that genetically determined early age at natural menopause is not causally associated with either CHD risk or with CHD risk factors [71]. Postmenopausal hormone therapy alleviates menopausal symptoms and results from observational studies consistently showed that the use of hormone therapy was associated with a lower risk of CHD and stroke [72, 73]. However, findings from RCTs on the effects of hormone therapy have been null or showed adverse effects on stroke risk. It now seems that timing is critical, and the benefits only seem to be present when the therapy is initiated temporally close to menopause and not when initiated later [74].

The effects of testosterone on health outcomes, in both women and men, are considerably less well-studied. A recent study in postmenopausal women, however, showed that the balance between testosterone and estrogens, as expressed by the testosterone/estradiol ratio, as well as testosterone levels per se, were associated with the risk of CVD [75]. Studies on the role of sex hormones in men’s health, although scarce, imply that higher levels of testosterone might be associated with a higher risk of CVD [76]. Also, sex hormone binding globulin (SHBG), which lowers circulating testosterone, might protect against CHD in men [65]. Future studies are needed to simultaneously assess the effects of multiple sex hormones, and their combinations, on a range of health outcomes in both women and men.

### Phase 3: Translation to policy and practice

In the first two phases of the roadmap for sex- and gender-disaggregated research, sex and gender differences are systematically identified and explained. The third phase focusses on the translation of the evidence obtained into policy and practice. Once those changes have been made, the actual uptake of policy and practice recommendations also needs to be evaluated. This is where implementation science plays a critical role. The field of implementation science seeks to systematically close the gap between what we know and what we do (often referred to as the know-do gap) by identifying and addressing the barriers that slow or halt the uptake of proven health interventions and evidence-based practices. As in all aspects of medical research, evidence for the efficacy (and potential disadvantages) of implementation is required. For example, cluster randomised trials can judge the merits of, for instance, training for awareness of unconscious sex bias or novel procedures designed to improve sex-specific diagnoses of stroke by ambulance crews. In the remainder of this section, we will discuss how evidence on sex and gender differences could be translated to policy and practice through education and clinical guidelines.

#### Education

Key to improving clinical practice will be to ensure that knowledge on known sex and gender differences, and the need to be sensitive to as yet unknown differences, is embedded into medical curricula, including for non-physician healthcare professionals. At present, however, most of the teaching around the impact of sex and gender on health focusses on the traditional aspects of women’s health; that is, sexual and reproductive health, and a broader view of how sex and gender as fundamental drivers of health and wellbeing is typically lacking. Even so, successful examples of implementing sex- and gender-inclusive medicine in medical curricula have emerged in several (mostly Western) countries, most notably Canada, Germany, and the USA [77]. Other countries, like Sweden, the Netherlands, and Korea, now also offer some form of sex- and gender-inclusive medicine in their curricula [78]. However, a shared characteristic of these initiatives is that they are often self-designated and driven by the vision and passion of a select group of individuals. As such, embedding of sex- and gender-inclusive medicine in education is still the exception, not the norm, in most parts of the world, and evaluations have yet to be done. To ensure the wide adoption of sex and gender in medical curricula, structural financial resources and commitment from the highest level of governmental or institutional leadership are essential [79].

#### Clinical guidelines

The results from sex- and gender-disaggregated research provide critical information to inform changes in clinical guidelines, which is the most direct way to change clinical practice. This could involve accounting for differences in prognosis between women and men and sex differences in access to, and uptake or effectiveness of medical interventions or health services. In the United States, women-specific guidelines for the primary prevention of cardiovascular disease were first released in 2003, with the latest update in 2019 [80]. These guidelines highlight the importance of female-specific risk factors, such as reproductive- and pregnancy-associated conditions in the future risk of CVD, as well as differences in manifestations and response to treatments. A review of 118 Canadian clinical practice guidelines published between 2013 and 2015 revealed that 35% contained sex-related diagnostic or management recommendations, 7% contained recommendations for sex-specific laboratory reference values, and 41% referred to differences in epidemiologic features or risk factors only [81]. A study in the Netherlands showed that guidelines on osteoporosis had the highest percentage of sex-specific recommendations (19%), whereas guidelines on depression had the lowest (none) [82].

In many fields, evidence may be insufficient to have sex-specific recommendations. In such cases, guideline committees should specify this upfront, as it informs practitioners about the scope of the guidelines and calls on the research community to provide the evidence required. Ensuring that guideline committees include an individual who is tasked to appraise the literature for evidence on relevant sex differences is key. Using a previously published framework for generating sex-specific guidelines [83], such an individual, with the support of the full writing committee, should systematically determine whether sex is relevant to the guideline and, if so, conduct a systematic appraisal of the included literature to determine whether sex-specific assessments of the quality of the evidence or the recommendations should be made. In clinical practice, the application of sex-specific recommendations, once available, will involve routinely asking whether the presentation, diagnostic workup, or management might change for each patient if they were the opposite sex. This might require a different cognitive mindset of clinicians, as many may not be familiar with this process. However, precision (or personalised) medicine is routine practice for many and thinking of the relevance of sex at different stages of preventative, diagnostic, and management process should just be part of it. Implicit bias assessment amongst the health care profession and research community would be one way of learning more about the problems [84].

### Systemic factors underpinning sex- and gender-disaggregated research

In order to systematically improve the uptake and quality of sex- and gender-inclusive research, women need to be better represented in clinical trials, funding and publishing successes need to depend on it, and academic leadership needs to be more diverse.

#### Representation of women in trials

Women remain underrepresented in RCTs [4, 5, 8, 10]. For example, while women account for nearly 50% of all CHD patients, they only account for about 25% of all participants in CHD trials [5]. The reasons underpinning this underrepresentation are unclear, but it may be that women are less likely than men to consider and/or to be considered for participation in trials. Data to support this assertion, however, are scarce and it is important to record, and publish, the reasons for non-participation in trials by sex, gender and other key socio-demographic variables, for example by conducting ‘studies within a trial’ [85].

Despite evidence to show the opposite, it is still frequently assumed that the evidence from these studies in (predominantly) male populations is equally applicable to women. For example, the Danish Cardiovascular screening trial (DANCAVAS) included an impressive number of 46,611 participants, but, disappointingly, all of them were male [86]. The assumption that the findings of a male-only trial can be directly translated to women is simply flawed and, in the case of pharmaceutical interventions, ignores fundamental differences between women and men in the pharmacokinetics and pharmacodynamics. Also, even when trials include women, they are often underpowered to reliably assess women-specific drug effectiveness, let alone sex differences in drug effects.

#### Academic funding and publishing

Routine conduct of sex- and gender-disaggregated research maximises the benefits of research for both women and men. However, such analyses are still often lacking in many medical disciplines, often without justification. Furthermore, women’s health journals should give better coverage of diseases that affect women’s health during the life course, including those that affect both sexes [87].

A range of interventions is likely to be needed to increase the uptake of sex- and gender-disaggregated research. Funders and publishers of medical research should make the integration of sex and gender a requirement for funding or publishing. If research not fulfilling this requirement simply does not get funded and/or published, this would rapidly change academic practices and would be a quick fix to the system. An excellent framework for evaluating the uptake of policies for integrating sex and gender, as well as other intersectional characteristics, into research design, has recently been published [2]. A growing number of funding agencies and academic journals already mandate that sex and gender are taken into consideration in research [2, 3, 88–90]. The Sex And Gender Equity in Research (SAGER) guidelines provide sound guidance on how sex and gender can be integrated in the design, analyses, and reporting of research [91] While these guidelines are increasingly being used, barriers to the uptake and implementation include concerns about mandating, and limited time, capacity, and resources, as well as their resistance or lack of awareness [92, 93]. A particular challenge is to assess adherence and to avoid this becoming a checkbox exercise. However, as with other editorial policies and research checklists, adherence to the SAGER guidelines should be an integral part of the publishing process. Improving knowledge about the importance of sex and gender in medical research within the research community is also likely to increase the uptake of such analyses. Excellent courses are available online and could increase awareness to such level to enable systemic change [94]. Including sex and gender champions in research teams would ensure that sex and gender are an integral part of research initiatives and would strengthen subsequent design, analyses, and reporting strategies. Over the past decade, the Canadian Institutes of Health Research has implemented multicomponent interventions to increase the uptake of sex and gender in applications for research funding. These interventions included mandatory reporting of sex and gender integration on applicant forms, development of resources for applicants and evaluators, and grant review requirements. A 10-year evaluation of these interventions not only showed a rise in the number of applications that integrated sex and gender, but also showed that applications that included sex and gender were also more likely to be funded [95]. An important next step would be to also assess whether these awarded projects genuinely conducted the sex and gender-disaggregated considerations they set out to do.

#### Diverse teams

A very pervasive factor, reaching far beyond the persistent lack of sex-and gender-disaggregated research alone, is implicit bias against women and the lack of women in leadership positions. Indeed, mounting evidence exists to show that a lack of gender balance can have wide-reaching negative consequences, including decreasing productivity, less innovation, and worse decision-making. The field of medicine is not an exception. Women are not only underrepresented as research participants, but also as producers and planners of research and in senior clinical roles [96–100] Research in the field of cardiovascular disease has shown that women attend conferences less frequently than their male colleagues, and if they attend, are less likely to speak or to attend as faculty. [97]. Gender bias is further exacerbated by the so-called child penalty, which, despite extension policies from funders, is a harsh reality for many, mostly, female academics. There is a wealth of data, however, showing that more women in different settings of academic research results in better science and more attention for sex and gender aspects in research. For example, greater representation of women in editorial boards is linked to a greater representation of women in key (i.e. first and last) authorship positions in various medical disciplines [101, 102], which in turn, is linked to a higher uptake of sex- and gender-based analyses [103]. Enhancing the diversity of teams reaches further than increasing sex and gender diversity alone. People from minority races and ethnicities, or from sexual minorities, also continue to be underrepresented or excluded. Men of minority races and ethnicities have also often been excluded.

Research benefits from including people from outside the academic community. Involving patients and the public throughout the research, from priority setting and planning to co-delivery and communication, allows for the inclusion of a broad range of voices and can enhance the quality and societal relevance of the research.

More diverse guideline committees are another critical component to ensure that the outcomes from sex- and gender-disaggregated research are translated into guideline recommendations and clinical practice [81, 83]. In doing so, ensuring that sex and gender are considered in guideline development becomes less of a task of a sex and gender champion alone. Indeed, diversification in both the clinical and scientific workforce and in the scientific studies is essential to produce the most rigorous and effective medical research. While the scale of the challenges may seem gigantic, a series of small steps made by individuals and institutions can lead to structural change and a more equitable world.

## Conclusions

Sex- and gender-disaggregated research and implementation are essential to ensure that women and men benefit equally from scientific progress. The field of sex- and gender-inclusive-based research is evolving and improving. Yet, the roadmap for sex- and gender-disaggregated health research presented here should remain relevant and outlines three basic phases that can aid researchers to systematically identify and explain relevant sex and gender differences, where they exist, and can aid educators, clinicians, and policymakers to translate the outcomes of research in the most effective and meaningful way.

## Data Availability

Not applicable.

## Abbreviations

CHD: Coronary heart disease
CVD: Cardiovascular disease
GWAS: Genome-wide association study
MR: Mendelian randomisation
RCT: Randomised controlled trial
SAGER: Sex and gender equity in research
SHBG: Sex hormone binding globulin
SNP: Single-nucleotide polymorphisms
